# Supplementary material for: Strained two-dimensional tungsten diselenide for mechanically tunable exciton transport
Source: Nat Commun. 2024 Dec 30;15:10847. doi: 10.1038/s41467-024-55135-8 (PMC11685721; doi:10.1038/s41467-024-55135-8)
Supplement: Supplementary file 1 — Supplementary Information [file 41467_2024_55135_MOESM1_ESM.pdf]

Supplementary Information

Strained two-dimensional tungsten diselenide for mechanically tunable exciton transport

Jin Myung Kim[1,2,†], Kwang-Yong Jeong[3,†], Soyeong Kwon[2,†], Jae-Pil So[4,5,†], Michael Cai Wang[6,7], Peter Snapp[6], Hong-Gyu Park[4], and SungWoo Nam[2,8*]*

[1]Department of Materials Science and Engineering, University of Illinois at Urbana-Champaign, Urbana, IL 61801, USA

[2]Department of Mechanical and Aerospace Engineering, University of California, Irvine, Irvine, CA 92697, USA

[3]Department of Physics, Chungnam National University, Daejeon 34134, Republic of Korea

[4]Department of Physics and Astronomy, and Institute of Applied Physics, Seoul National University, Seoul 08826, Republic of Korea

[5]Department of Physics, Soongsil University, Seoul 06978, Republic of Korea

[6]Department of Mechanical Science and Engineering, University of Illinois at Urbana-Champaign, Urbana, IL 61801, USA

[7]Department of Mechanical Engineering, University of South Florida, Tampa, FL 33620, USA

[8]Department of Materials Science and Engineering, University of California, Irvine, Irvine, CA 92697, USA

*Corresponding Authors (Emails: sungwoo.nam@uci.edu, hgpark@snu.ac.kr)

†These authors contributed equally to this work.

Methods

(1) Sample fabrication

We prepared monolayer tungsten diselenide (WSe_2) flake via mechanical exfoliation from single crystalline bulk WSe_2 (HQ Graphene, Netherlands). Specifically, we used 300 nm-thick SiO_2/Si wafers as exfoliation substrate. Before exfoliation, polyacrylic acid (PAA) film (about 100 nm thickness) was deposited on SiO_2/Si substrate as a sacrificial layer for wet transfer by spincoating 5 wt% of polyacrylic acid (PAA) solution (MW ~ 50000, Polyscience Incorporated) for 60 s at 5000 rpm. After finding 1L-WSe_2 flakes via optical contrast and photoluminescence (PL) spectrum, polymethyl methacrylate (PMMA) (950PMMA A2, MicroChem) was spincoated on WSe_2/PAA/SiO_2/Si for 30 s at 6000 rpm. Separately, we prepared a polydimethylsiloxane (PDMS) substrate by mixing base elastomer and curing agent (Sylgard 184, Dow Corning Incorporation) with 10:1 weight ratio and curing at 70 °C for 60 min. Then, we prestretched a PDMS substrate with 20% of prestrain, followed by oxidizing PDMS surface with oxygen plasma treatment for 180 s at 150 mTorr pressure and 250 W of plasma power to form a stiff silica skin layer on top of PDMS. To transfer WSe_2 onto silica/PDMS substrate, PMMA/WSe_2 was separated from the wafer by floating it on the surface of DI water by dissolving the PAA layer. The separated PMMA/WSe_2 film was cleaned by DI water and transferred to the prestretched silica/PDMS substrate. After drying it in ambient condition for more than 48 hours, we slowly removed prestrain of PDMS. Detailed fabrication process is illustrated in Supplementary Fig. 1a. For two-point bending measurement, we transferred PMMA/WSe_2 on 500 μm-thick polycarbonate (PC) substrate.

(2) Structural and optical characterization

Geometry of wrinkle structure was characterized by atomic force microscope (AFM) (Cypher, Asylum Research, CA, USA) and 3D laser scanning confocal microscope (VK-ZX1000, Keyence, Japan). The thickness of oxidized silica layer on PDMS was measured by X-ray reflectivity (XRR) (PANalytical Philips X'pert MRD diffractometer, USA) using Cu K α radiation (λ = 0.154056 nm)[1]. Photoluminescence (PL)/Raman point spectra and line profiles were obtained by Raman confocal imaging microscope (LabRAM HR, Horiba, Japan) and PL area map was measured by confocal Raman microscope (Nanophoton Raman 11, Nanophoton Corporation, Japan) equipped with line illumination. All the PL/Raman measurements were performed with 532 nm excitation laser. Time-resolved PL (TRPL) and pump-probe measurements were carried out using a home-built fiber-coupled confocal microscope (Supplementary Fig. 7). The setup was composed of two sets of two-dimensional galvo mirror scanning systems in order to control pump and probe positions respectively. A pulsed red laser (632 nm) was used for excitation in TRPL measurement and a continuous green laser (532 nm) was used for excitation in pump-probe measurement. The beam was focused through a 100x objective with a numerical aperture (NA) of 0.9. The detection signal was focused into a single mode fiber. A fiber splitter then led the coupled signal either to a spectrometer (Acton SpectraPro™, Princeton Instrument Inc.) for spectral measurements or to an avalanche photodiode (APD; Excelitas SPCM AQRH 13). The time-correlated-single-photon-counting (TCSPC, PicoHarp300) was used to obtain the time resolved PL. For low-temperature PL measurement, the sample was mounted in a vibration-isolated cryostat cooled by compressed helium and a 50x microscope objective lens (NA 0.42) was used for confocal PL setup similar to the room temperature measurement.

Supplementary Text

(1) Strain prediction from mechanical model of wrinkle structure

Surface-instability driven wrinkling is induced by macroscopic compression of stiff thin film on soft substrate. Since our wrinkle structure is composed of several thin film layers, including silica skin layer, 1L-WSe_2, and encapsulation PMMA layer, theoretical calculation of local strain on wrinkled WSe_2 requires numerical simulation with relevant mechanical and interfacial information. Here we used a simple model of linear elasticity theory of sinusoidal wrinkles with one layer component. In this calculation, the maximum tensile/compressive strain of the wrinkled film is proportional to the thickness of the film and local curvature. We assume that (1) wrinkles follow periodic sinusoidal shape, (2) the effect of PMMA is negligible due to relatively lower adhesion force to WSe_2, (3) strong adhesion at both silica/PDMS and silica/WSe_2 interfaces. The maximum strain at the top/bottom of the wrinkles is expressed as below[2,3],

$$\varepsilon_{\max} \sim \pi^2 \frac{h_s + h_w}{1-\nu_{eff}^2}\frac{\delta}{\lambda^2} \qquad (1)$$

where ε_{max} is maximum tensile/compressive strain at the apex/valley of the wrinkle, h_s is thickness of silica skin layer, h_w is thickness of monolayer WSe_2, ν_{eff} is effective Poisson's ratio of silica/WSe_2 layer, δ is height of the wrinkle, and λ is wavelength of the wrinkle. Since the plate theories in continuum mechanics are not suited to estimate strain applied on atomically-thin membranes due to atomic thinness and low bending rigidity, we focus on the maximum strain applied at the apex/valley of silica skin layer and assume perfect strain transfer between silica skin layer and WSe_2 (assumption (3)). This assumption requires further justification by analyzing interfacial adhesion and excluding slippage/delamination at the interface, which is beyond the scope of this paper. Nevertheless, perfect strain transfer has been assumed in a wide range of strain analysis of 2D materials, particularly when it comes to 2D materials supported on a

substrate. One example is to apply uniform tensile strain on 2D materials by placing 2D flake on thick polymer substrates (e.g., polycarbonate, polyethylene terephthalate glycol, polyvinyl alcohol) and conformally bending 2D/polymer substrate. Uniform strain was calculated with simple bending model ($\varepsilon = t / 2R$, where t is thickness of 2D/polymer and R is radius of curvature). This model assumes (1) complete strain transfer between polymer and supported 2D material, (2) strain-free state of as-fabricated flat sample, and (3) no lateral strain gradient in 2D material. This method has been widely adopted for various types of 2D materials (graphene, MoS_2, $MoSe_2$, WSe_2, WS_2) to measure strain-tunable properties of 2D materials[4–10].

To apply the equation for strain estimation, we measured the thickness of silica layer, which was estimated to be ~12 nm by X-ray reflectivity measurement (Supplementary Fig. 1d) and fitting into a bilayer model (silica/PDMS) using Motofit software[11] for scattering length density calculation with respect to the depth from the silica surface[1]. The changes in scattering length density underneath the surface is attributed to varying electron density in bilayer system, and the thickness of silica layer is determined by measuring depth at which scattering length density is 5% above the plateau (PDMS). Effective Poisson's ratio is calculated based on Poisson's ratio of each layer (0.17 for silica, 0.19 for WSe_2) and their relative thickness ratio. The height and wavelength of the wrinkles are obtained by 3D confocal laser profile (Fig. 1b). Using these parameters, we calculated ε_{max} to be 0.69%. It agrees well with compressive strain at the valley (0.73%), while tensile strain at the apex is approximately twice higher than the estimated value (1.47%). This is explained by the smaller radius of curvature at the apex (Supplementary Fig. 1c) and strain inversely proportional to the radius of curvature in the linear elasticity theory.

(2) The role of neutral excitons and lattice strain in tuning optical responses in wrinkled WSe_2

As shown in Fig. 1c, the highly asymmetric PL was observed under local tensile (apex) and local compressive (valley) strains, different from more symmetric PL spectrum observed from uniform tensile strain (Supplementary Fig. 10). The asymmetry of the room-temperature PL emission spectrum of monolayer WSe_2 is usually observed due to phonon scattering of momentum indirect excitons. The energy gradient within the excitation area and funneling effect possibly enhance the asymmetric PL in case of local strain engineering.

To further elucidate the asymmetry of PL emission, we performed low-temperature PL measurement of the flat and wrinkled WSe_2 prepared using the same fabrication condition (Supplementary Fig. 16-19). The unstrained flat WSe_2 showed relatively weak and blueshifted PL emission of A exciton at low temperature compared to that measured at room temperature (E = 1.74 eV) (Supplementary Fig. 16). In addition, we observed several peaks emerged at low temperature, which may be ascribed to reduced thermalization and increased probability to form low-energy, weakly-bound excitonic components such as trion (1.71eV)[12], biexciton (1.68 eV)[13], and/or localized/bound excitons (<1.68 eV)[14,15]. These trends of temperature-dependent PL emission were not affected in the regions of neutral exciton and trion with presence of a PMMA encapsulating layer, indicating minimal effect of polymer capping. We note that PL peaks with energies below 1.65 eV exhibit minor differences at less than 100 K depending on the presence of PMMA, whereas such differences tend to disappear as increasing temperature (Supplementary Fig. 17).

Meanwhile, we observed strong PL emission along apex lines of the wrinkled WSe_2 at low temperature (Supplementary Fig. 18). The observed PL emission peaks in wrinkled WSe_2 may

be attributed to dense ensemble of localized excitons by exciton funneling at the wrinkle apex[15] with reduced phonon scattering at low temperature. However, such sharp emission of localized exciton was observed only at low temperature (Supplementary Fig. 16) because of thermal detrapping of bound excitons at elevated temperature.

At cryogenic temperatures (Supplementary Fig. 16a), a weak peak is observed from strained WSe_2 at an energy of 75 meV lower than the relatively distinct X^0 peak of unstrained WSe_2, suggesting that this is the weakened neutral exciton of strained WSe_2. It is noteworthy that at room temperature (Supplementary Fig. 16b), the X^0 peak in strained WSe_2 shows a similar red-shift of 75 meV compared to unstrained WSe_2. In addition, we can see the X^0 peak blue-shifted by 78.5 meV at 15 K compared to the X^0 peak at 298 K. As the temperature decreases, lattice contraction occurs and the electron-phonon interaction decreases. Consequently, the energy band gap increases, resulting in the central wavelength of the neutral excitons to be blue-shifted in the range of 100 meV[16]. The neutral exciton peaks weaken at cryogenic temperatures due to enhanced radiative pathways from defect states. This, alongside the consistent neutral exciton peak shift (75 meV) compared to room temperature, supports our observation of the dominant presence and contribution of neutral excitons at room temperature.

The observation of exciton funneling, even at low temperatures (Supplementary Fig. 19), displays a similar pattern to the pump-probe PL maps at room temperature as a function of the excitation position (Supplementary Fig. 9). Specifically, when the excitation laser is positioned at the apex, emission predominantly arises from the apex. In contrast, when the excitation laser is set at the valley, emission is still probed from the apex region due to exciton funneling toward the apex.

It provides compelling evidence of strain gradient-induced bandgap modulation, which enhances PL intensities and redshifts the PL energy peaks at the apex. The less prominent features of funneling at low temperatures are attributed to the fact that either (1) the enhanced radiative recombination channels of localized defect sites at cryogenic temperatures may inhibit the drift of neutral excitons, or (2) the dominant localized excitons at these temperatures may not drift as efficiently as neutral excitons. These results suggest that PL enhancement with a red shift of the emission peak at the apex in locally strained WSe_2 at room temperature are unlikely dominated by other excitonic components. Instead, these effects may be driven by strain gradient-induced changes in the neutral A exciton, such as an energy gradient in the excitation area or exciton funneling.

To investigate governing role of neutral excitons at room temperature in the wrinkled WSe_2, we carried out differential reflectance spectroscopy (Supplementary Fig. 20). In general, the optical absorption spectrum derived from the differential reflectance resonates with direct transitions, providing more comprehensive information on charge transitions, which is advantageous for measuring the energies absorbed by electrons during interband transitions[17–19]. In contrast, PL primarily reflects indirect transitions and is more sensitive to radiative recombination during the transition to the lowest energy state, thereby revealing localized defects and other excitonic complexes along with neutral excitons.

To this end, white light illumination from a halogen lamp was directed to the wrinkle sample through an iris aperture and then the reflected light was collected by confocal setup equipped with galvo-mirror scanning system. The differential reflectance spectra were recorded from unstrained flat WSe_2 and wrinkled WSe_2 at apex and valley (Supplementary Fig. 20a-c). For the flat WSe_2, PL and reflectance presented similar energy for A exciton at around 1.66 eV whereas

reflectance revealed the B exciton at 2.1 eV due to spin-orbit splitting at the valence band.[20] In the case of wrinkled WSe_2, reflectance spectra were red- and blue-shifted at the apex and valley, respectively, compared with that of unstrained, flat WSe_2 (similar to the observation in PL spectroscopy), indicating that PL and reflection arose from the same excitonic transition.

Furthermore, the spatially-resolved hyperspectral linescan images of PL and 2nd derivative of differential reflectance (Supplementary Fig. 20d-f) reveal distinct changes in the PL and reflectance peaks along the distance from the apex. It should be noted that the energy difference between PL and reflectance observed for our wrinkled WSe_2 is less than 6 meV, which is distinct from the energy shift of other excitonic components such as charged trions (30 meV),[21] biexcitons (50-60 meV),[22] or defect-localized excitons (>60 meV).[23,24] The energy difference observed between reflectance and emission is typically attributed to the Stokes shift, where the emitted wavelength is longer than the absorbed wavelength due to energy loss. For WSe_2, the Stokes shift is reported to be around 3 meV[25], while our WSe_2 wrinkled structure shows a slightly larger but still minimal shift of 6 meV, suggesting minimal defect influence on the strained WSe_2.

While intrinsic defects may naturally form in WSe_2 and could be deconvoluted in the PL peak at room temperature, we strongly believe that these defects do not significantly affect our observations of neutral excitons funneling in room temperature via strain-induced band gap modulation. As it has been demonstrated, the alignment in peak position observed in differential reflectance and PL further supports the notion that room temperature PL in this work is substantially dominated by neutral A excitons originating from the direct transitions.

(3) Estimation of exciton funneling length

The exciton funneling length can be expressed as below[26],

$$l = \left\langle v_{drift} \tau_{rad} \right\rangle = \frac{\nabla E}{m_r} \tau_{dephase} \tau_{rad} \sim \frac{\Delta E}{m_r l} \tau_{dephase} \tau_{rad} \quad (2)$$

$$l = \sqrt{\frac{\Delta E}{m_r} \tau_{dephase} \tau_{rad}} \quad (3)$$

where l is funneling length, $\langle v_{drift} \rangle$ is average drift/funneling velocity, τ_{rad} is radiative lifetime, E is exciton energy, m_r is reduced mass of exciton, and $\tau_{dephase}$ is optical dephasing time. In theoretical calculation reported earlier, exciton funneling length of nanoindented monolayer MoS_2 was estimated to be about 3 μm. Based on our experimental parameters and reported physical values (ΔE = 118 meV, m_r = 0.2 m_e[27], $\tau_{dephase}$ = 0.25 ps[28], τ_{rad} = 1.5 ns), we calculate the effective funneling length to be 6.2 μm. Because this estimation does not consider dark-to-bright exciton conversion and drift-induced optical darkening of bright exciton, it underestimates the possible exciton funneling length, implying that 2.5 μm of funneling length in our observation is reasonable within theoretical bound.

(4) Exciton decay simulation

The continuity equations for bright (KK) excitons (4) and momentum-forbidden dark (D) excitons (5) excitons are:

$$\frac{\partial n_b}{\partial t} = -\frac{n_b}{\tau_b} - \eta n_b^2 + D\nabla^2 n_b + \mu_b \gamma_b \nabla\left(n_b \left(\nabla \varepsilon\right)\right) - C_{bd}\left(n_b - n_d \exp\left(\frac{\Delta E_{bd}}{k_B T}\right)\right) \quad (4)$$

$$\frac{\partial n_d}{\partial t} = -\frac{n_d}{\tau_d} - \eta n_d^2 + D\nabla^2 n_d + \mu_d \gamma_d \nabla\left(n_d \left(\nabla \varepsilon\right)\right) + C_{bd}\left(n_b - n_d \exp\left(\frac{\Delta E_{bd}}{k_B T}\right)\right) \quad (5)$$

where subscripts b and d refer to bright and dark excitons respectively, n is the exciton density, t is time, τ is exciton lifetime, η is the exciton-exciton annihilation constant, D is diffusivity, μ is

mobility, γ is the strain sensitivity of exciton energy, ε is applied strain, C_{bd} is the dark-to-bright exciton conversion coefficient, ΔE_{bd} is the energy difference between bright and dark excitons, k_B is the Boltzmann constant, and T is temperature. Accurate estimation of the parameters in equations (4) and (5) is necessary to insure the validity of our numerical solution.

Beginning by accounting for our experimental conditions, we can determine appropriate modeling functions for applied strain, ε, and exciton density, n, by examining our extracted data. ε was estimated from the PL shift allowing us to set local strain at the apex and valley of wrinkles. For calculation of the strain gradient, $\nabla\varepsilon$, the strain profile across wrinkle structure was approximated as having an ~$\sin^4(\frac{\pi x}{\lambda})$ form, where λ is wrinkle wavelength, based on the steep strain gradient near apex than valley in PL/Raman profile (Fig. 1e). In the case of flat structures strain is assumed to be uniformly 0%. Excitation at t = 0 is assumed to follow 2D Gaussian distribution (σ^2 = 0.2 μm^2) and generate equal number of bright and dark excitons[29]. Having accounted for experimental conditions we can move onto estimation of physical parameters for generated excitons. For both bright and dark excitons, the exciton-exciton annihilation constant, η (0.36 cm^2 s^{-1})[30], and exciton diffusivity, D (2 cm^2 s^{-1})[31], are assumed to be identical. Concerning parameters which diverge between bright and dark excitons, bright exciton behavior is well reported allowing us to extract bright exciton mobility, μ_b (300 cm^2 V^{-1} s^{-1})[32], and strain sensitivity of energy, γ_b (49 meV/%strain)[33], from the literature. In contrast, dark exciton mobility, μ_d, and strain sensitivity of energy, γ_d, have not been explicitly reported, requiring estimation from the values for bright excitons. The mobility of Q-valley charge transport is less than 1/20 of K-valley mobility due to strong phonon scattering at Q-valley, resulting in negligible contribution of dark exciton drift[34]. Similarly, the strain sensitivity of dark excitons, $\gamma_{d,}$

in monolayer WSe_2 is approximately one fifth of that of bright excitons, γ_b, with opposite sign, allowing estimation of γ_d (e.g., $\gamma_d / \gamma_b = -0.22$ (uniaxial strain)[33] or -0.25 (biaxial strain)[35]).

In addition to inherent exciton behavior, we need to account for exciton lifetime, τ. Focusing on bright excitons, the delay in radiative recombination of bright excitons due to drift momentum is given by (6)[36].

$$\tau_b(Q) = \frac{\tau_b(0)}{1-\left(\frac{cQ}{E(Q)}\right)^2} \qquad (6)$$

where Q is exciton momentum, $\tau_b(Q)$ is radiative lifetime of bright exciton with momentum Q, and c is speed of light. For exciton decay time with zero drift-momentum (i.e., flat WSe_2), we obtained three exponential components from the TRPL curves in Fig. 3a and assumed a radiative decay lifetime, τ_b, of 1.5 ns at room temperature. Drift momentum was derived from drift velocity according to the Drude model. For the non-radiative decay of dark excitons (τ_d), there are no reported experimental or theoretical estimations, but the ratio of non-radiative decay of spin-forbidden dark excitons to radiative decay is reported to be more than 50[37], implying much slower decay of optically-forbidden transition state compared to radiative direct transition. In our calculation, we assumed ten times longer decay time of non-radiative, momentum-forbidden dark exciton vs. radiative decay time.

Finally, it is possible for dark excitons to convert to bright excitons requiring us to determine the dark-to-bright exciton energy difference, ΔE_{bd}, and the dark-to-bright exciton conversion coefficient, C_{bd}. We chose $\Delta E_{bd}(\varepsilon=0) = -60$ meV based on recent first principles calculations[38]. Energy difference at nonzero strain, $\Delta E_{bd}(\varepsilon)$, was determined from $\Delta E_{bd}(\varepsilon=0)$ by correcting for local strain using the strain sensitivity of bright/dark excitons (γ_b/γ_d). The phonon scattering rate

for dark-to-bright conversion (C_{bd}) has not been reported yet, so was used as a fitting parameter for recreation of the TRPL curves.

Using the above parameters, we solved (4) and (5) numerically using Mathematica software and the results are plotted in Supplementary Fig. 12. All the parameters are identically applied for simulation of pump-probe emission maps, exciton flux, and conversion rate in Fig. 3g-i.

It should be noted that our calculation mainly elucidates exciton motion as functions of diffusion and drift, but the model is established based on the quantum effect of excitons, such as conversion between indirect dark excitons and direct bright excitons in momentum space. In addition, strain-induced exciton transport and localization is closely related to exploring quantum mechanical phenomena and functionalities by increasing density of localized cold excitons in Bose-Einstein condensation and antibunched recombination of localized excitons (single photon emitters). Thus, our model may pave a pathway for rational design of quantum straintronic devices based on strain engineering of atomically-thin semiconductors.

(5) Dielectric screening and doping effects from the encapsulating layer and the skin layer

Light-matter interactions in atomically-thin semiconductors are strongly affected by dielectric environment. Our system consists of WSe_2 supported on a silicon oxide layer, which induces strong dielectric screening effects and thus leads to weaker exciton binding energy and reduced quasiparticle bandgap compared with those of freestanding WSe_2. Here, we consider two dielectric screening factors from amorphous silica skin layer. First, dielectric screening of underlying silica layer is determined by high frequency dielectric constant. Since the dielectric constant of silicon oxide is reported to be 2.1[39,40], it may induce less stronger dielectric screening effect than h-BN (4.5)[39] or sapphire (4.1)[41]. However, it should be noted that dielectric

screening does not significantly alter the energy of 1s state exciton (i.e., A exciton) in WSe_2 at room temperature, while the optical bandgap of higher excitonic states (2s to 4s) decreases at higher dielectric screening environment[41]. Because the exciton transport described in this manuscript focuses on energy gradient effect on A exciton, the dielectric screening of silica layer may have limited contribution to exciton transport in terms of substrate material selection.

The other aspect we might need to consider is the gradient factor arising from local strain gradient. The local strain exerted to silica can change local dielectric constant and cause variation of dielectric screening effect. However, strain-induced tuning of the dielectric constant of amorphous silica layer requires significant degree of stress (i.e., 450 MPa of biaxial stress for 1% of variation in dielectric constant)[42]. Considering maximum local tensile strain at the apex of silica layer (<3%) and the Young's modulus of silica (70 GPa), the local variation in dielectric constant is estimated to be less than 3%. Thus, the influence of local variation in dielectric constant of silica layer is expected to be small in exciton transport, in comparison to energy modulation induced by local strain on WSe_2.

In addition to the dielectric screening effect, there could be doping effect due to contact with a polymer layer. In this work, we used PMMA (thickness of ~70 nm) as an encapsulating material, to avoid the undesirable effects of h-BN that may appear in our wrinkle system. For example, the use of thick and stiff h-BN (typical thickness of >30 nm) on both sides or top side of monolayer WSe_2 can cause further disturbed wrinkling because the stiffness of h-BN (~860 GPa for monolayer and few-layer h-BN[43]) is much higher than that of soft polymer (~3 GPa for thin film PMMA[44]). In fact, when h-BN was used, we often found that no wrinkles were formed around h-BN or bubbles were trapped at the interface between h-BN and WSe_2. Furthermore, we examined various substrates, including SiO_2, PMMA and h-BN, and measured PL emission of

WSe_2 (Supplementary Fig. 21). The measurement showed that the PL peak emission wavelengths were almost same at ~750 nm (1.65 eV), which corresponds to the intralayer emission of WSe_2[33]. In addition, considering similar peak wavelengths and emission shapes, we can conclude that there is no substantial doping effect or exciton funneling effect from the PMMA substrate compared with SiO_2 or h-BN.

(6) Exciton routing simulation

Our exciton routing model is based on checkerboard-shape wrinkle structure which exerts biaxial strain on the supported WSe_2. The checkerboard shape wrinkle is superposition of perpendicular uniaxial wrinkles propagating in x and y directions, which has been experimentally demonstrated by using surface-instability driven wrinkling process, similar to our uniaxial wrinkling, in several previous reports[45–48]. Its geometry is similar to that of MoS_2 supported on egg-box shape 3D rigid nanostructure[49], but checkerboard shape wrinkling using soft PDMS substrate allows for mechanically reconfigurable strain tuning and exciton routing. We intend to control exciton transport direction as a function of mechanically reconfigurable local strain. More specifically, Fig. 4e shows that excitation at the valley of the structure creates high energy excitons at t = 0 ns and biaxial local strain causes exciton drift toward (1,1) direction, if there is no external stretching in x- and y- directions. By changing local strain (e.g., stretching) in x- and y- directions we can induce exciton drift to (0,1) and (1,0) directions, respectively. The input and output signals are incident photon and emitted photon, respectively, identical to the electrically-driven exciton routers. In our model, dynamic on/off switching is not considered in the frequency calculation because local strain gradient is kept static after reconfiguration by external strain.

Unlike uniaxial wrinkle structure, our model involves biaxial strain and shear strain on WSe_2. The compatibility conditions for strains in 2D system can be described in the below equation[50]:

$$\frac{\partial^2 \varepsilon_{xx}}{\partial y^2} + \frac{\partial^2 \varepsilon_{yy}}{\partial x^2} = 2\frac{\partial^2 \varepsilon_{xy}}{\partial x \partial y} \qquad (7)$$

Similar to the tensile/compressive strain, the shear strain can modulate the band structure and affect exciton funneling if there is shear strain gradient. For instance, the shear strain sensitivity of MoS_2 A exciton is theoretically calculated to be -50 meV/%strain, which is similar to uniaxial strain sensitivity of the same A exciton (-49.4 meV/%strain)[51]. To take the shear strain into account for our exciton transport model, we adopt a 2D thin plate model. The thin plate consists of silica layer and monolayer WSe_2 (thickness *t*), the same as our uniaxial wrinkle structure. Because of the checkerboard shape wrinkle structure, we assume 2D sinusoidal deformation of the thin plate, which is described by the multiplication of sinusoidal function in x- and y-direction ($w = A\sin(2\pi x/\lambda)\sin(2\pi y/\lambda)$), where *A* is proportionality constant and λ is distance from a peak to nearest peak.[52]. The bending-induced tensile/compressive strains and shear strain applied on WSe_2 are derived by below equations in the small-deflection theory:

$$\varepsilon_{xx} = -\frac{t}{2}\frac{\partial^2 w}{\partial x^2} \qquad (8)$$

$$\varepsilon_{yy} = -\frac{t}{2}\frac{\partial^2 w}{\partial y^2} \qquad (9)$$

$$\varepsilon_{xy} = -\frac{t}{2}\frac{\partial^2 w}{\partial x \partial y} \qquad (10)$$

The strain distribution of the checkerboard wrinkle structure is plotted in the Supplementary Fig. 22a-b. To determine the exciton energy modulation from the strain map, it is necessary to consider the strain sensitivity of exciton energy for direct biaxial strain and shear strain. For the biaxial strain, Theoretical study has demonstrated that biaxial strain sensitivity of monolayer

WSe_2, A exciton energy is approximately twice higher than uniaxial strain sensitivity ($\gamma_{uniaxial}$ = 57.6 meV/%, $\gamma_{biaxial}$ = 115.2 meV/%)[53]. On the other hand, the shear strain sensitivity of A exciton of monolayer WSe_2 has not been demonstrated theoretically or experimentally. It is reported that shear strain on monolayer MoS_2 modulates exciton energy by ca. 16 meV/%shear strain for small shear strain (< 2%) which is approximately one-third of uniaxial strain sensitivity of monolayer MoS_2[54]. Based on these theoretical predictions, we assumed that the biaxial strain sensitivity and shear strain sensitivity of monolayer WSe_2 is twice and one-third of the uniaxial strain sensitivity, respectively. Then we calculated the exciton energy distribution map in the basis of checkerboard wrinkle strain and strain sensitivity (Supplementary Fig. 22c). In this model, the distance between nearest peaks is 5 μm and the biaxial strain difference between peak and valley is 1.5%. We simulated exciton transport in the checkerboard wrinkle WSe_2 with the same physical parameters of A excitons used in the uniaxial wrinkle calculation.

Supplementary Figure

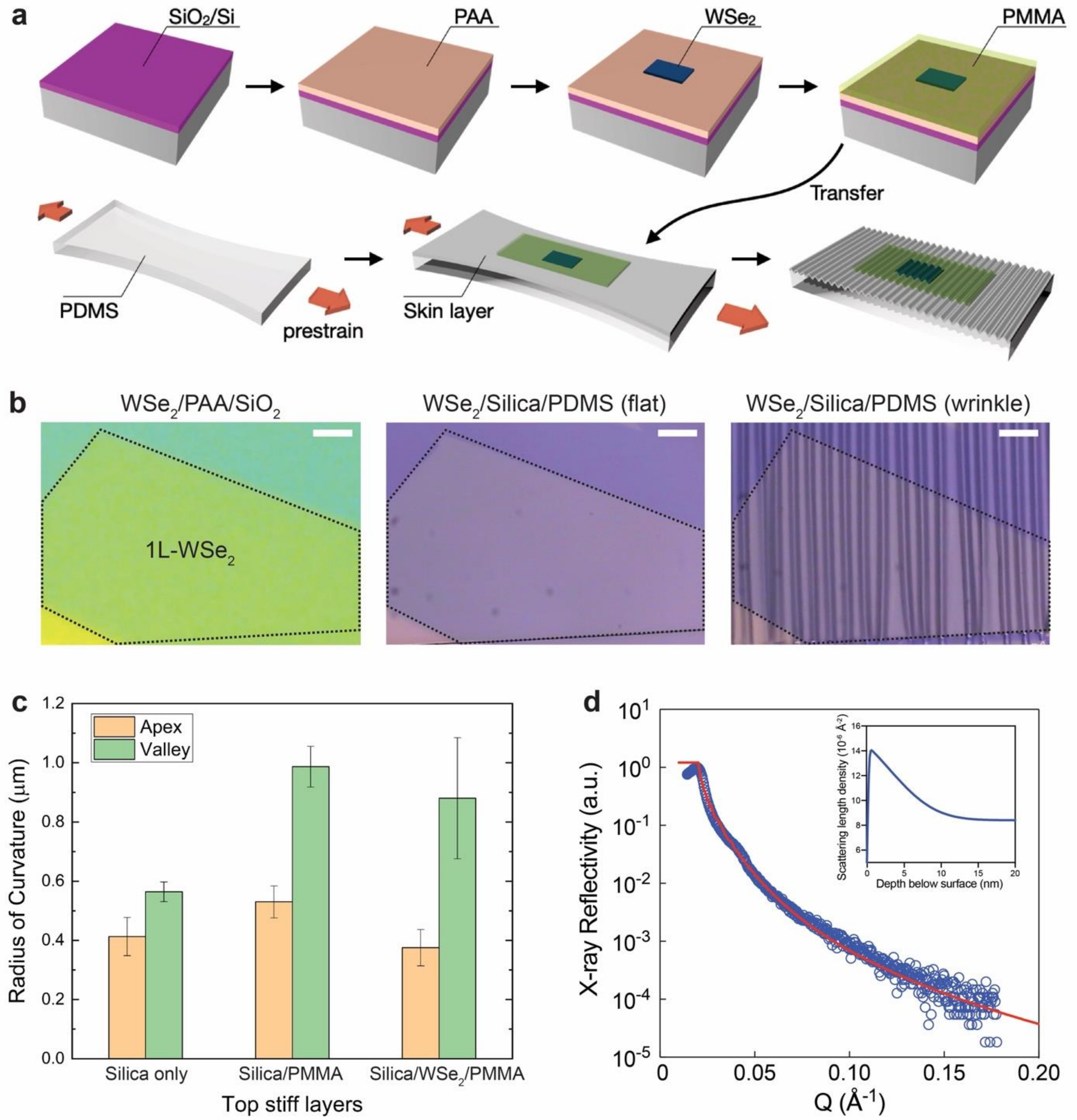

Supplementary Fig. 1. Fabrication process and structural analysis of wrinkle architecture with monolayer WSe_2. a, Schematic illustration of fabrication steps of wrinkled WSe_2. **b,** Optical microscope image of as-exfoliated WSe_2 on PAA-coated SiO_2/Si wafer (left), transferred but unreleased WSe_2 on silica/PDMS (center), and prestrain-released WSe_2 with wrinkle structure (right). **c,** Change in radius of curvature of wrinkles at apexes and valleys. Error bars

indicate one standard deviation. **d,** Characterization of thickness of silica skin layer via X-ray reflectivity. Silica layer was formed on a flat PDMS substrate, with the same O_2 plasma treatment condition. The measured reflectivity data (blue dots) are fitted to bilayer model in analysis software (Motofit). The inset plot is the calculated scattering length density as a function of depth below surface.

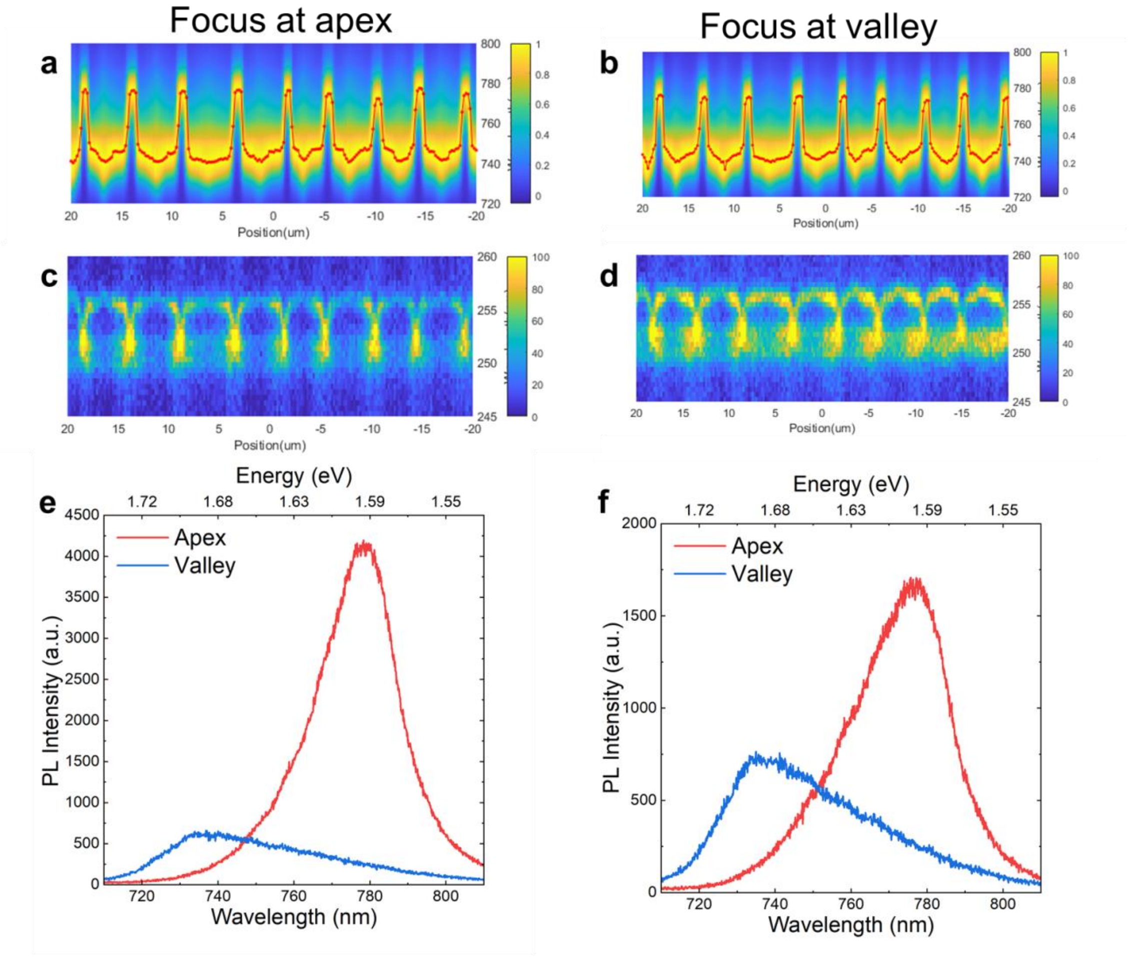

Supplementary Fig. 2. Effect of focal plane shift in PL/Raman measurement. PL/Raman line scan and point spectra was obtained as a function of laser focus either at apex or valley. **a,c,e,** Excitation laser focused at apex. **b,d,f,** Focused at valley. When laser is focused at apex, there is relative enhancement of both PL and Raman intensities at apex than valley due to outfocusing of incident laser at valley. On the contrary, focusing laser at valley resulted in overall similar Raman intensities at apex and valley due to influences of geometry/curvature variation as well as laser outfocusing at apex. However, PL enhancement at apex occurs despite valley-focusing with similar Raman intensities, implicating possible exciton funneling toward apex under localized tensile strain.

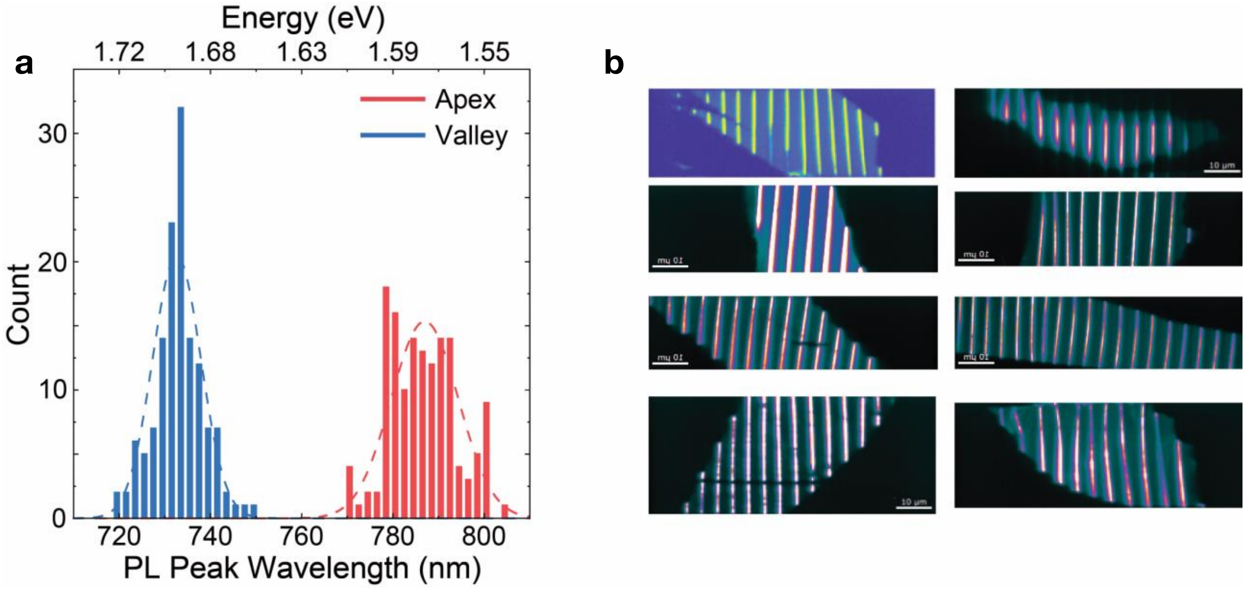

Supplementary Fig. 3. Statistical analysis on PL shift of wrinkled WSe_2. a, Histogram of strain-induced PL shift in 15 different samples with 147 apexes and 140 valleys. Dashed lines are Gaussian fitting of histogram data, showing average energy at apex and valley of 1.574 ± 0.014 eV and 1.693 ± 0.012 eV, respectively. **b,** PL intensity maps of selected wrinkle samples at the PL peak wavelength of apex.

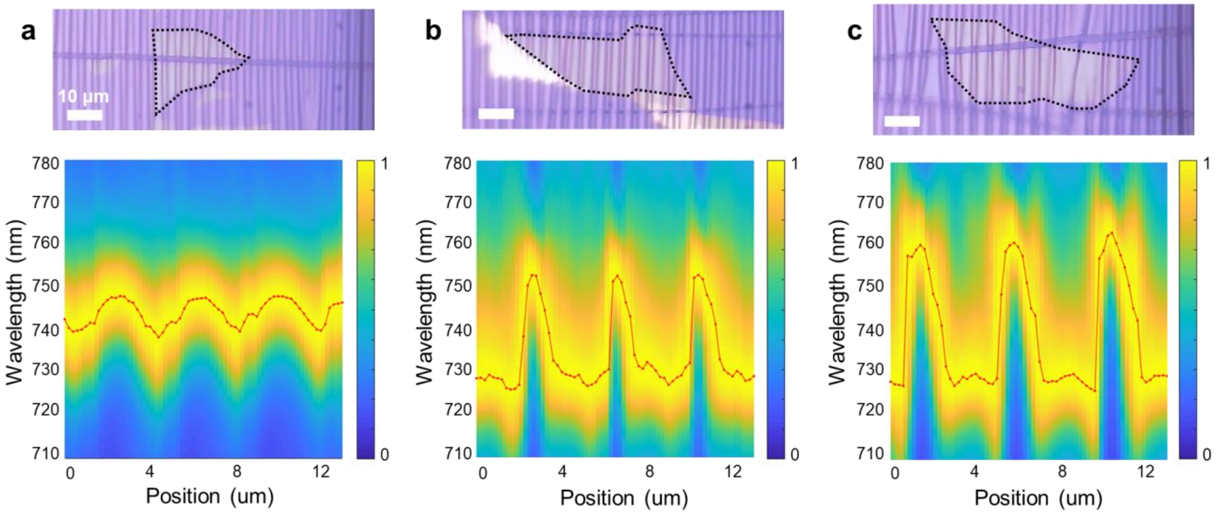

Supplementary Fig. 4. Tunable wrinkle architecture by changing thickness of silica skin layer. a-c, Different oxygen plasma treatments for 1 min (**a**), 2 min (**b**), and 3 min (**c**) induce silica layer on PDMS to be 11.1 nm, 12.1 nm, and 14.7 nm estimated by X-ray reflectivity analysis.

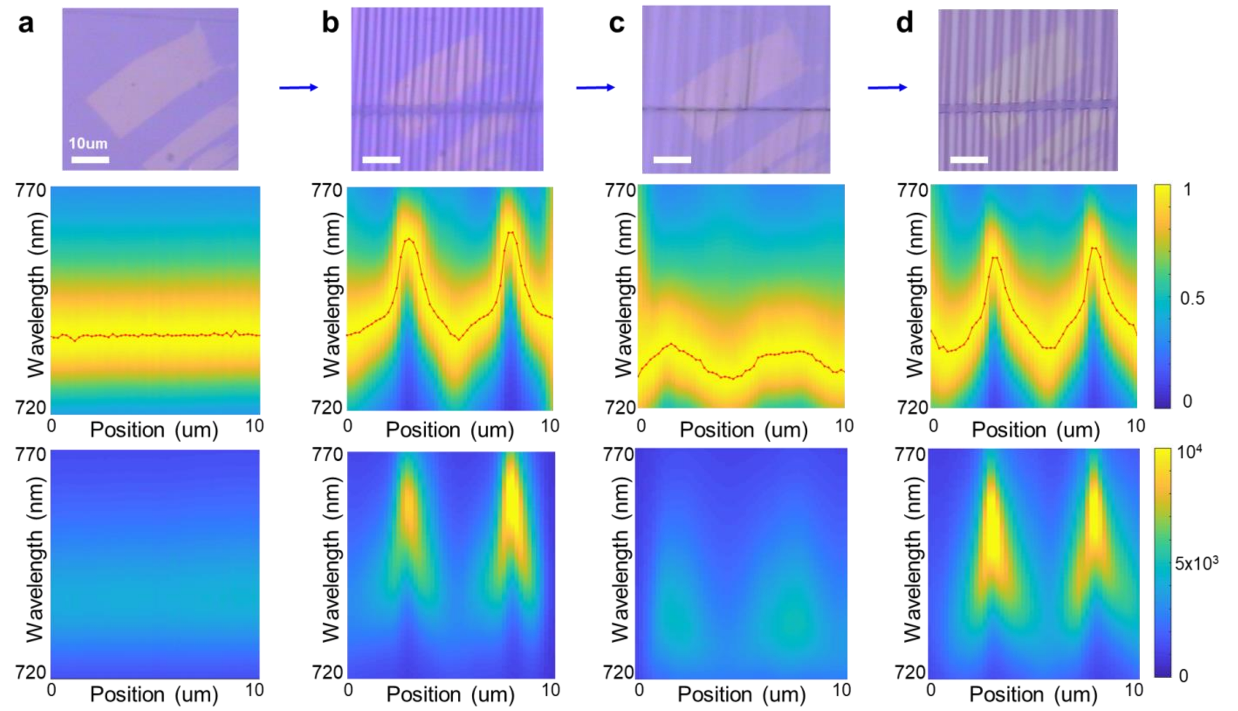

Supplementary Fig. 5. Mechanically reconfigurable strain control of conformally wrinkled WSe_2. a, Flat WSe_2 with prestrain unreleased. **b,** Initial wrinkle structure after the prestrain released. **c,** Restretched state where external strain is about 90% of prestrain. **d,** Releasing external strain again to form wrinkle structure. The normalized PL line map reveals strain modulation as the wrinkle structure restretched and released again. The absolute PL line map shows PL intensification at apex due to strain-induced exciton funneling effect.

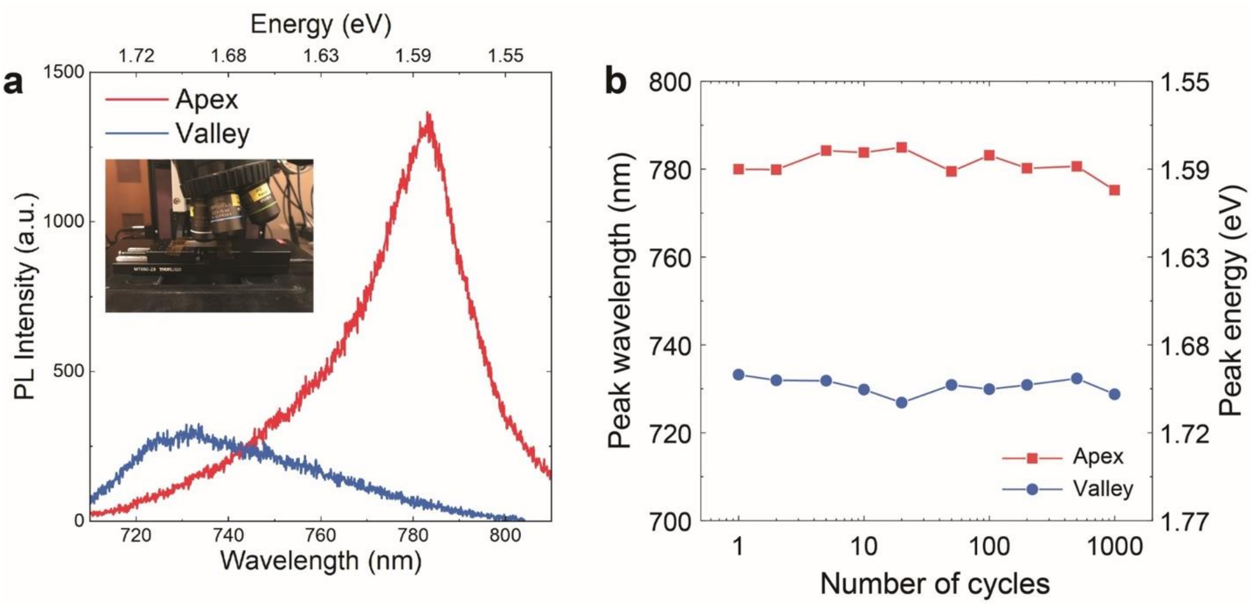

Supplementary Fig. 6. Cyclic measurement of mechanical reconfigurability. a, PL emission spectra measured at the apex and valley before cyclic test. Inset image shows PDMS substrate mounted on an automated translation stage and loaded for PL measurement. **b,** PL peak wavelength (left y-axis) and corresponding peak energy (right y-axis) at apex (red) and valley (blue) were recorded over 1000 cycles of restretching (50% of initial prestrain) and releasing processes. Approximately 92% of initial local strain maintained after 1000 cycles.

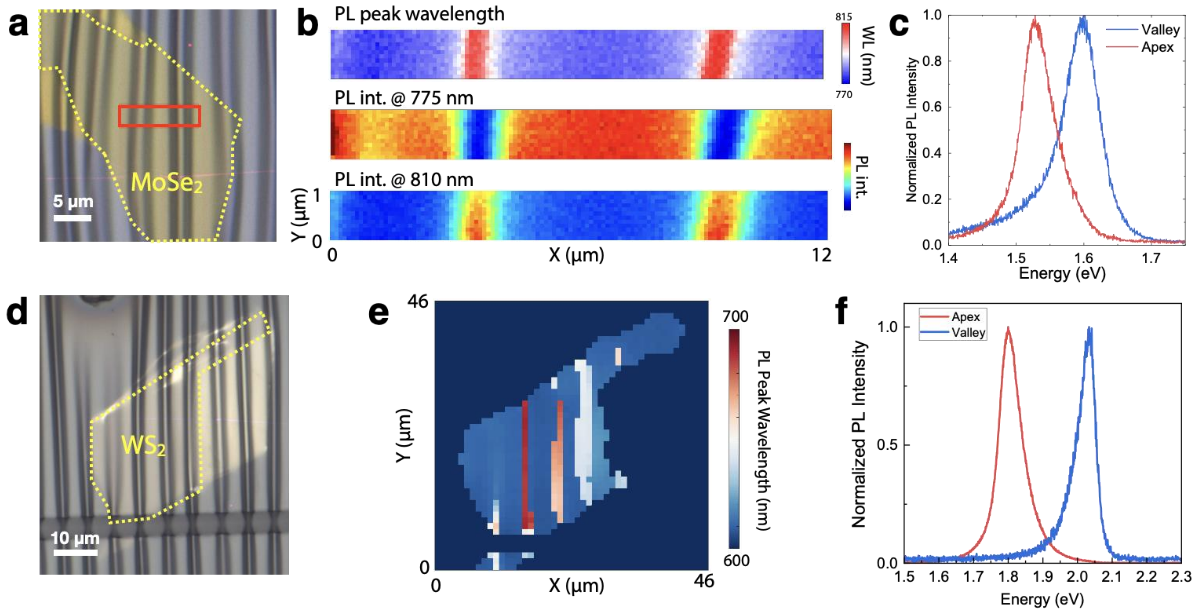

Supplementary Fig. 7. Wrinkle structure prepared with different transition metal dichalcogenides monolayers. a, Optical microscope image of wrinkled monolayer $MoSe_2$. b, Hyperspectral imaging in the red squared regime in a. (top) PL peak wavelength map, (middle) PL intensity map for 775 nm wavelength emission. (bottom) PL intensity map for 810 nm emission. c, PL emission spectra of wrinkled $MoSe_2$. d, Optical microscope image of wrinkled monolayer WS_2. e, PL peak wavelength map obtained by hyperspectral PL imaging. f, PL emission spectra of wrinkled WS_2.

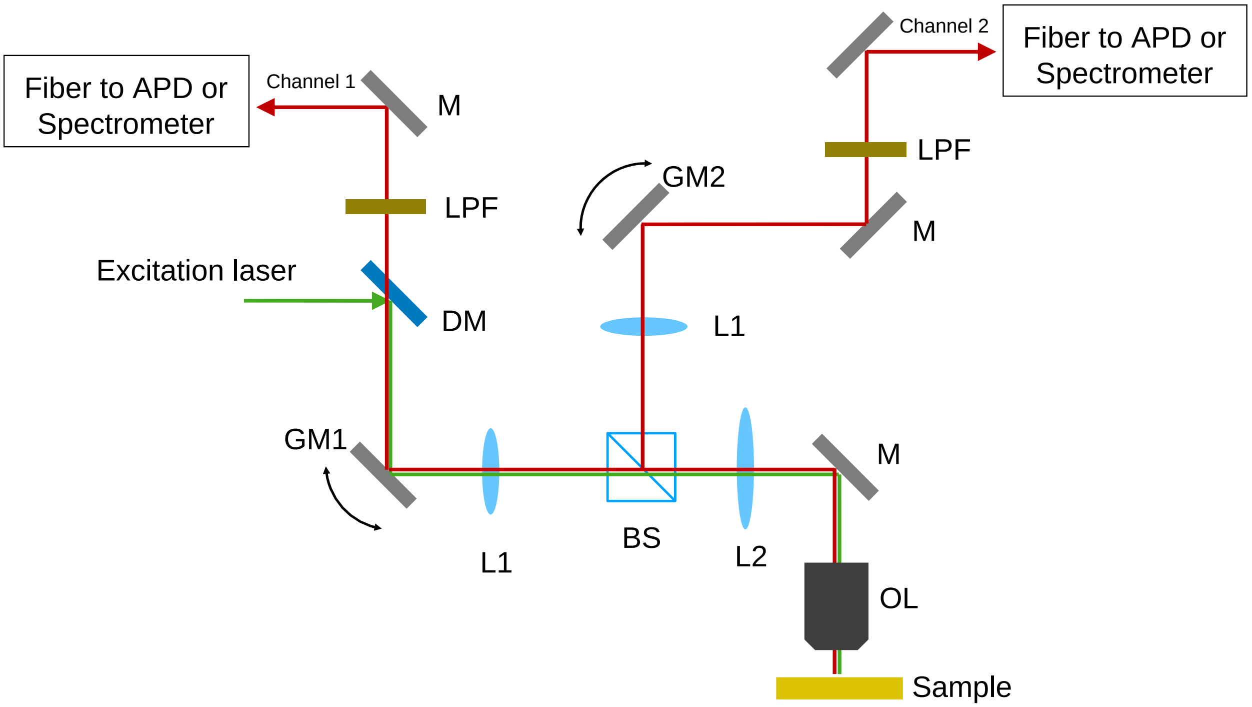

Supplementary Fig. 8. Confocal pump-probe measurement setup. M: Mirror, GM1 and GM2: Galvo mirrors, L1 and L2: Lens, BS: Beam splitter, DM: Dichroic mirror, OL: Objective lens, LPF: Long pass filter.

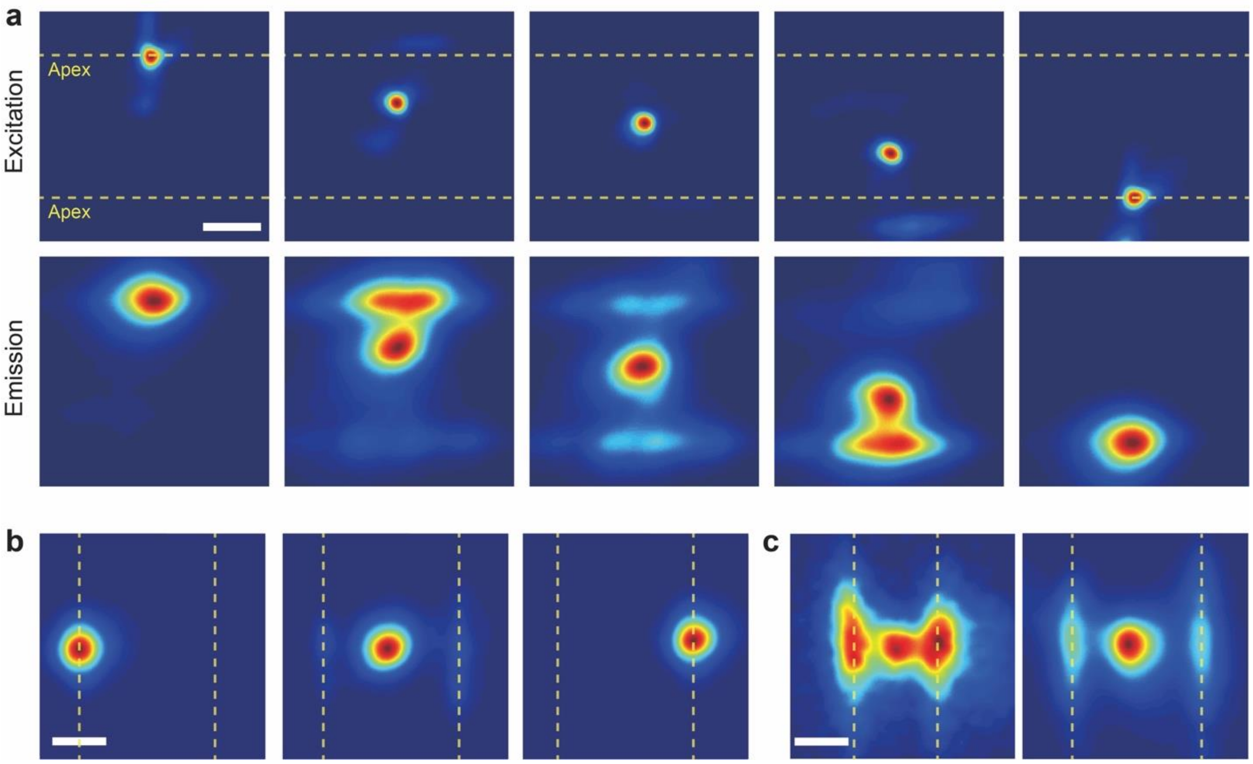

Supplementary Fig. 9. Pump-probe PL maps as a function of pumping position. a, Measurements of sequential excitation from one apex to the adjacent apex (top) and corresponding emission maps (bottom). **b,** PL emission map of weakly strained WSe_2 (ε= 0.8%) with excitation point fixed at the left apex (left), the center valley (middle), and the right apex (right), showing less apparent funneling effect. **c,** PL emission map of WSe_2 with shorter funneling distance (d ~ 1.5 μm) and longer funneling length (2.9 μm, right apex). Scale bars, 2 μm. The pump-probe excitation/emission maps were image-processed by 2D Gaussian filtering in MATLAB to compensate the background noise signal, while the raw images in Fig. 2d result in the same trend.

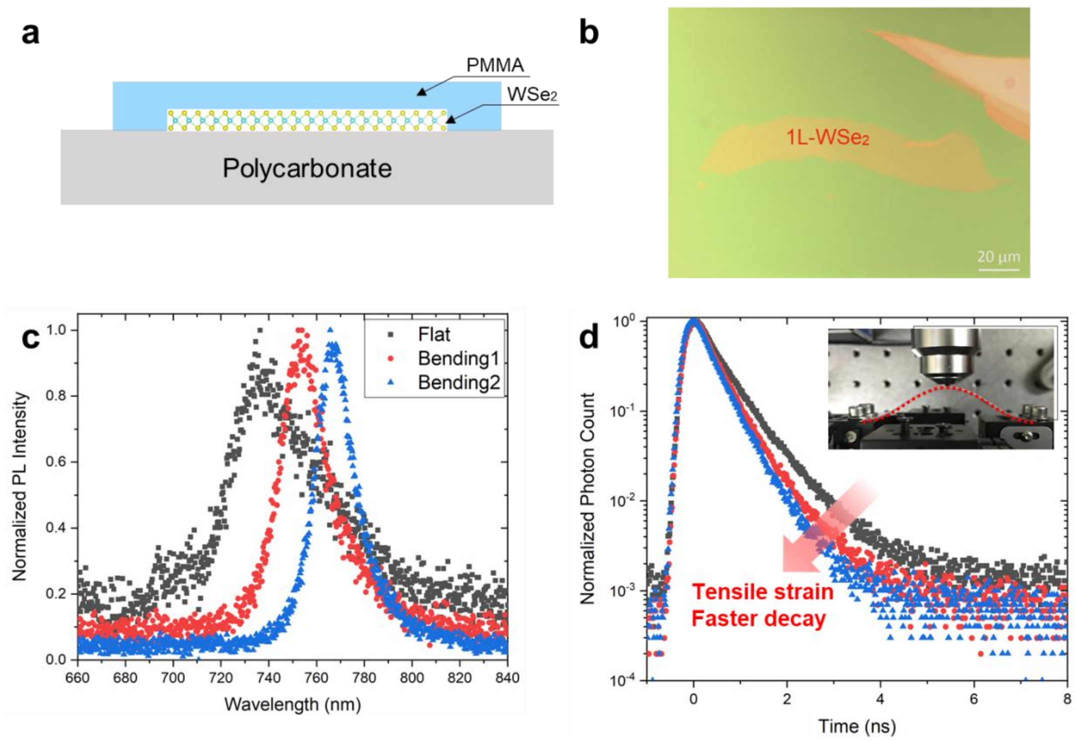

Supplementary Fig. 10. Spatially homogeneous strain and time-resolved PL decay. a, Bending sample prepared by wet transfer of PMMA-supported WSe_2 on oxygen plasma treated polycarbonate (PC) substrate. **b,** Optical microscope image of monolayer WSe_2 on PC. **c,** PL shift as WSe_2 is bent. **d,** Time-resolved PL curve with different bending tensile strain with inset figure showing bending curvature.

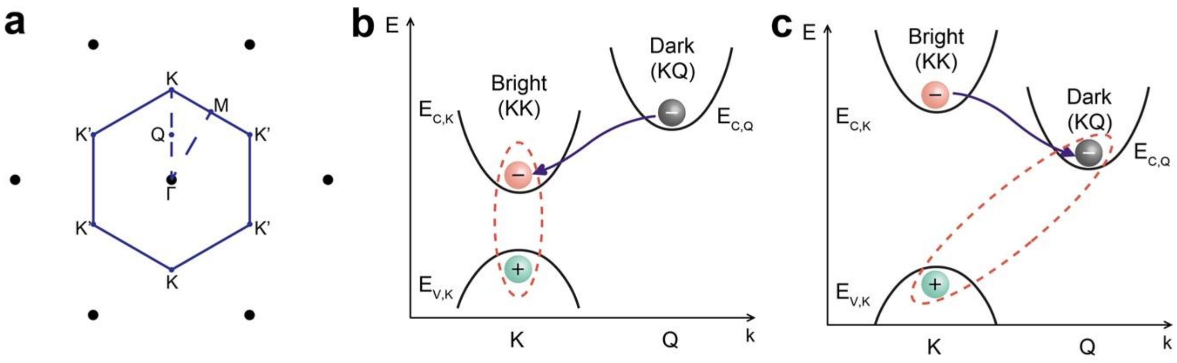

Supplementary Fig. 11. Schematic illustration of momentum space of wrinkled WSe_2. a, First Brillouin zone points of unstrained WSe_2 in momentum space. **b**, Band diagram at the apex of wrinkled WSe_2, with lower energy of direct KK excitons. **c**, Band diagram at the valley of wrinkled WSe_2, showing lower stability of bright exciton compared to momentum-indirect dark exciton.

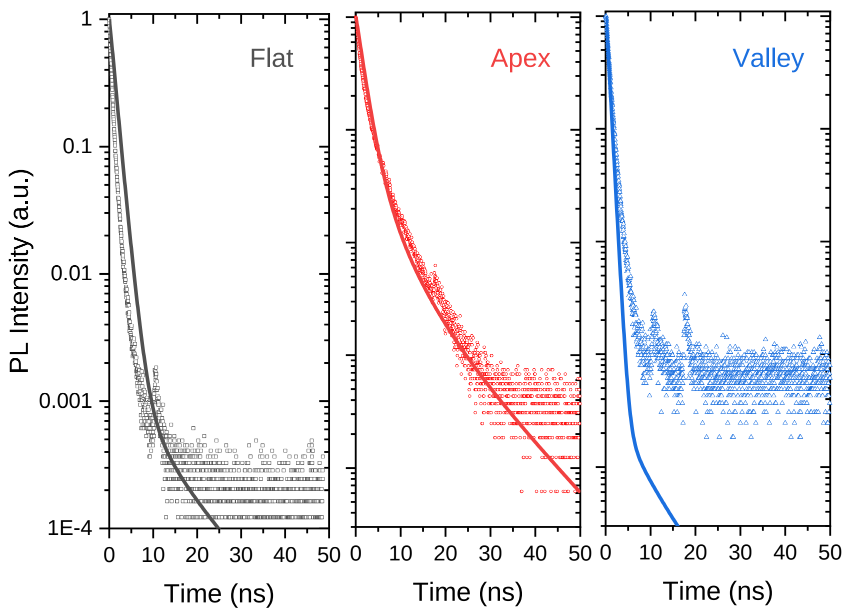

Supplementary Fig. 12. Experimental and simulated TRPL from flat and wrinkled WSe_2. Experimental TRPL curves are denoted as dots and simulated decay curves are the bold line based on our exciton continuity equation.

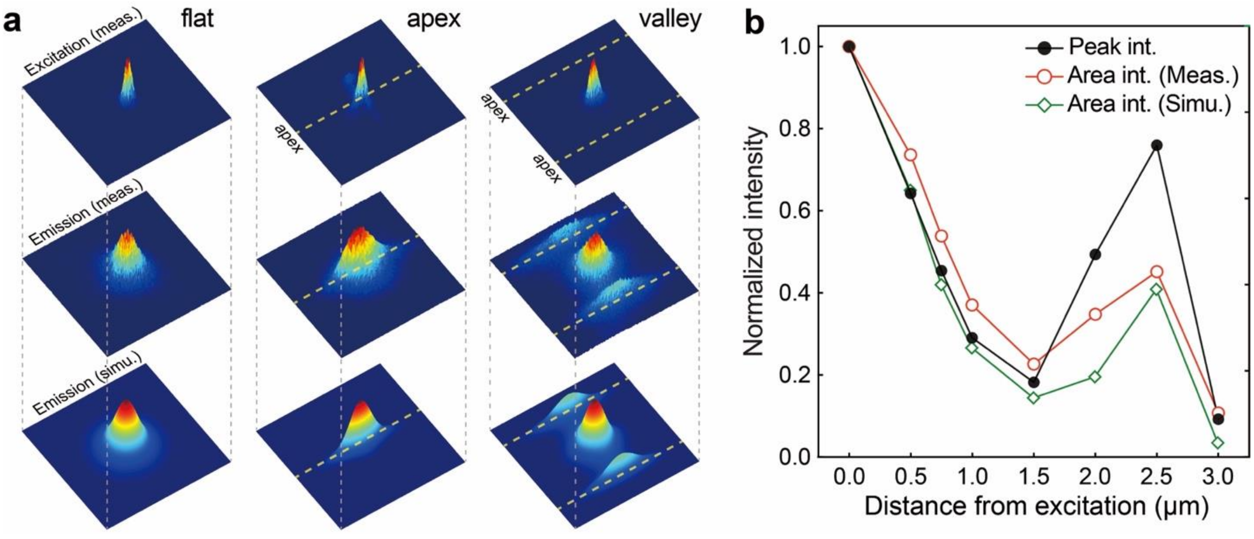

Supplementary Fig. 13. Comparison of measured and simulated pump-probe PL maps. a, Measured excitation maps (first row), measured emission maps (second row), and simulated emission maps (bottom) from flat WSe_2 (left) and wrinkled WSe_2 excited at apex (center) and valley (right). **b,** Emission characteristics of funneling excitons in terms of measured PL peak intensity (black), measured fluorescence (area integrated PL; red), and simulated fluorescence (green).

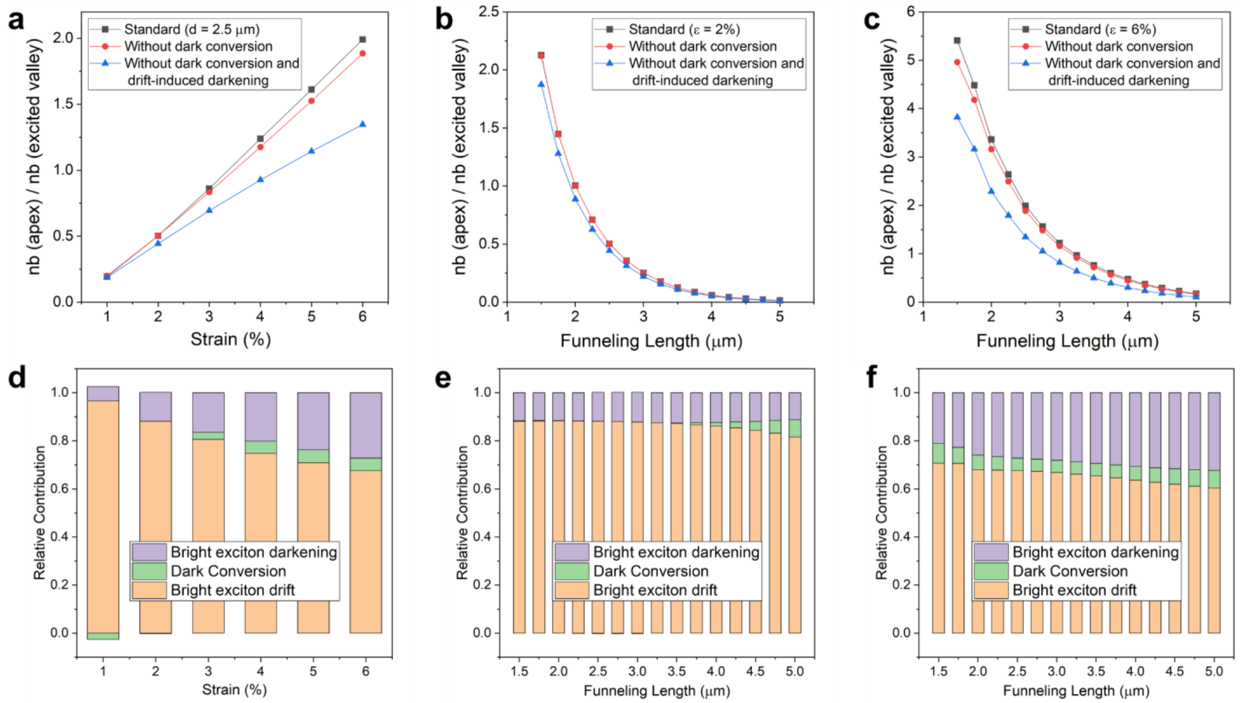

Supplementary Fig. 14. Simulated exciton density at apex relative to excited valley with and without consideration of dark-to-bright exciton conversion and/or drift-induced darkening of bright excitons. **a-c,** Funneled exciton density with respect to applied strain (a) and funneling length (b, c). Funneling length in **a** is 2.5 μm and applied strain in **b** and **c** is 2% and 6%, respectively. **d-f,** Contribution of bright exciton drift, dark-to-bright exciton conversion, and drift-induced darkening of bright exciton to exciton density at apex in the cases of **a-c**.

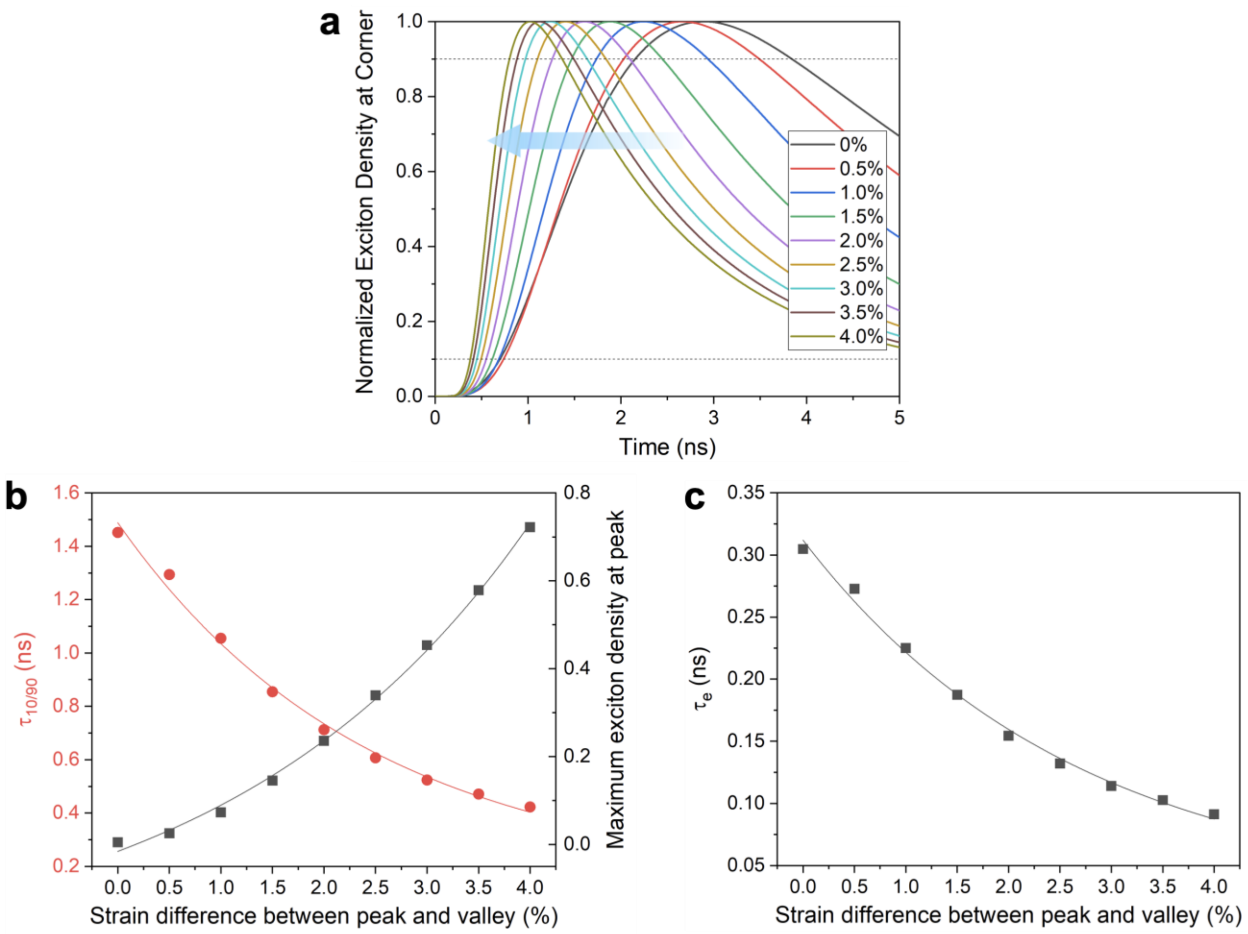

Supplementary Fig. 15. Simulation of transient exciton drift of biaxially strained WSe_2 under different strain level. a, Normalized exciton density at (1,1) position in Fig. 4b as a function of detection time and strain. Average strain gradient for both x- and y-direction varies from 0% to 2.5%. The dotted lines indicate 10% and 90% of normalized exciton density at probing position, which was used to calculate $\tau_{10/90}$. **b,** Summary of 10/90 rise time ($\tau_{10/90}$) and maximum exciton density at (1,1) position. Increasing strain enables faster exciton transport and greater signal obtained at the probing position. **c,** Exponential time constant at the rising edge (τ_e) as a function of strain gradient between (1,0) and (0,0).

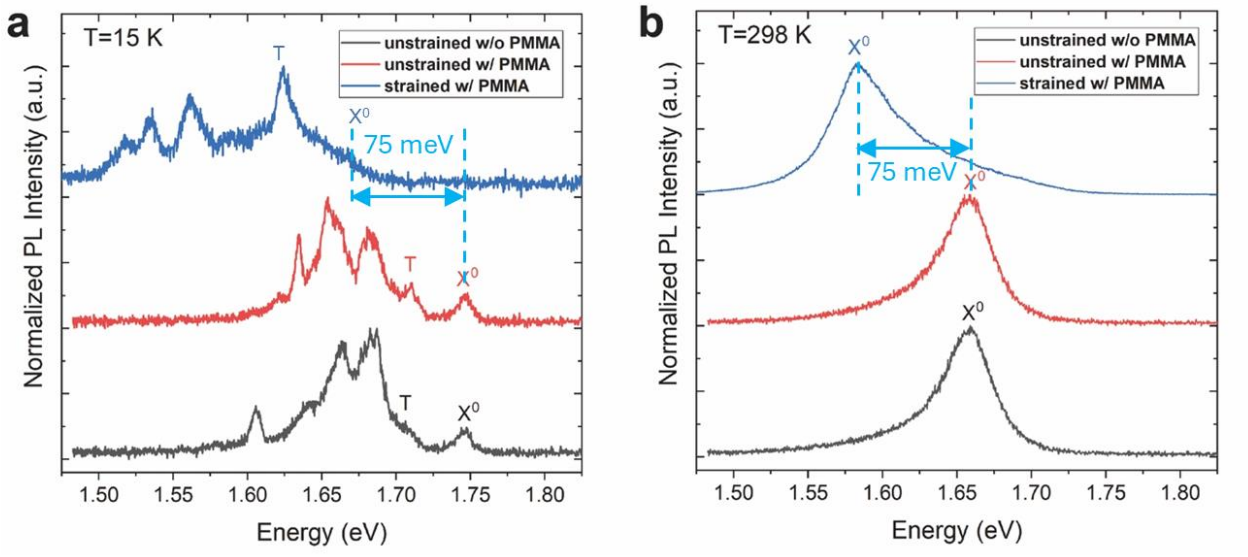

Supplementary Fig. 16. PL emission of unstrained/strained WSe_2 with and without PMMA thin film at varying temperature. a, PL emission spectra measured at 15 K. **b**, PL emission spectra measured at 298 K. Neutral exciton (X^0) and trion (T) were indexed in the plot. For the unstrained PL emission, we used the same monolayer WSe_2 flake with and without PMMA coating at both temperatures, while we employed different strained WSe_2 flakes due to wrinkle formation.

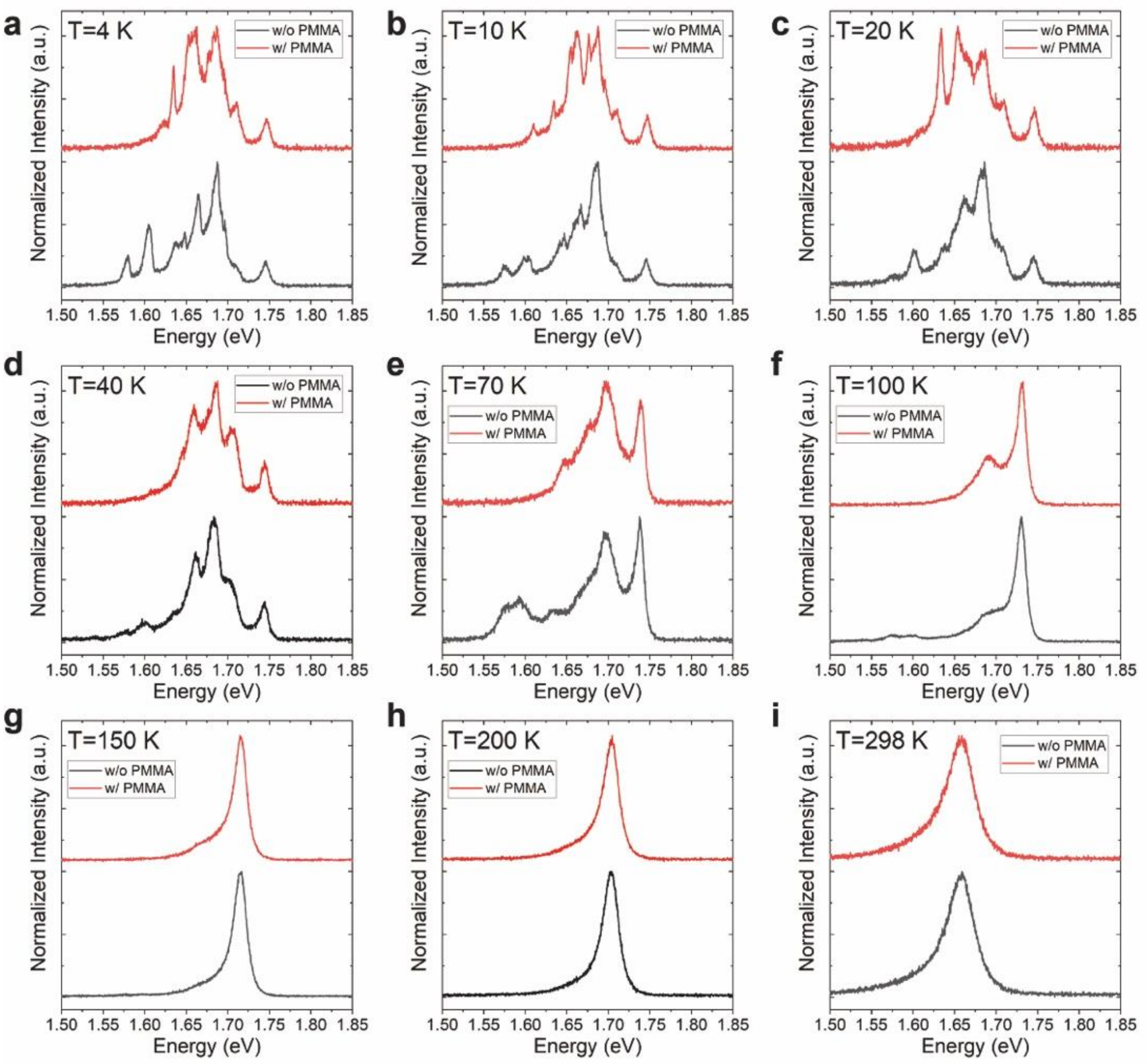

Supplementary Fig. 17. PL emission of unstrained WSe_2 with and without PMMA thin film at varying temperature. PL measurement was carried out at (a) 4 K, (b) 10 K, (c) 20 K, (d) 40 K, (e) 70 K, (f) 100 K, (g) 150 K, (h) 200 K, and (i) 298 K.

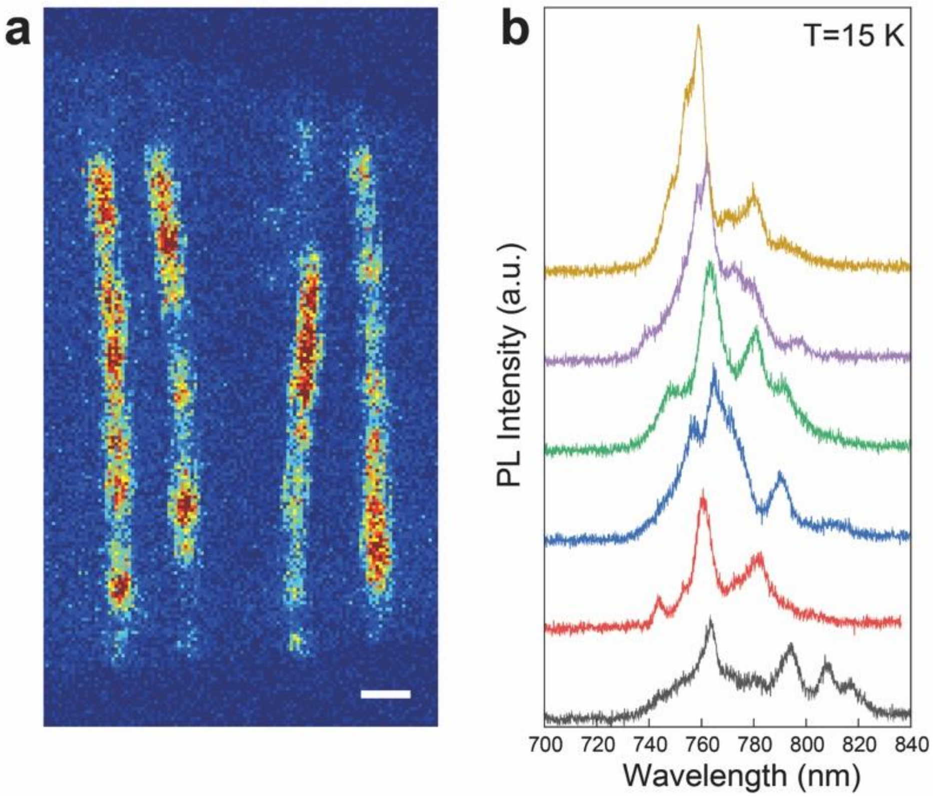

Supplementary Fig. 18. Enhanced PL emission of strained WSe_2 at low temperature. a, Fluorescence intensity map of wrinkled WSe_2, showing localized strong PL emission along the apex line. The scale bar is 5 μm. **b,** PL spectra of wrinkled WSe_2 at localized emission points in (a) at T = 15 K.

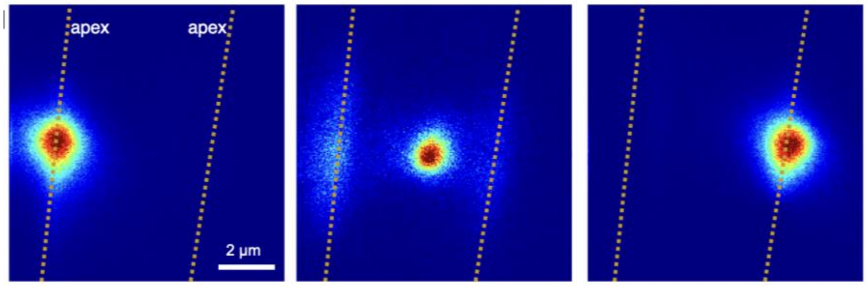

Supplementary Fig. 19. Demonstration of exciton funneling at cryogenic temperature. Pump-probe scanning maps with excitation laser fixed at left apex, valley, and right apex, respectively.

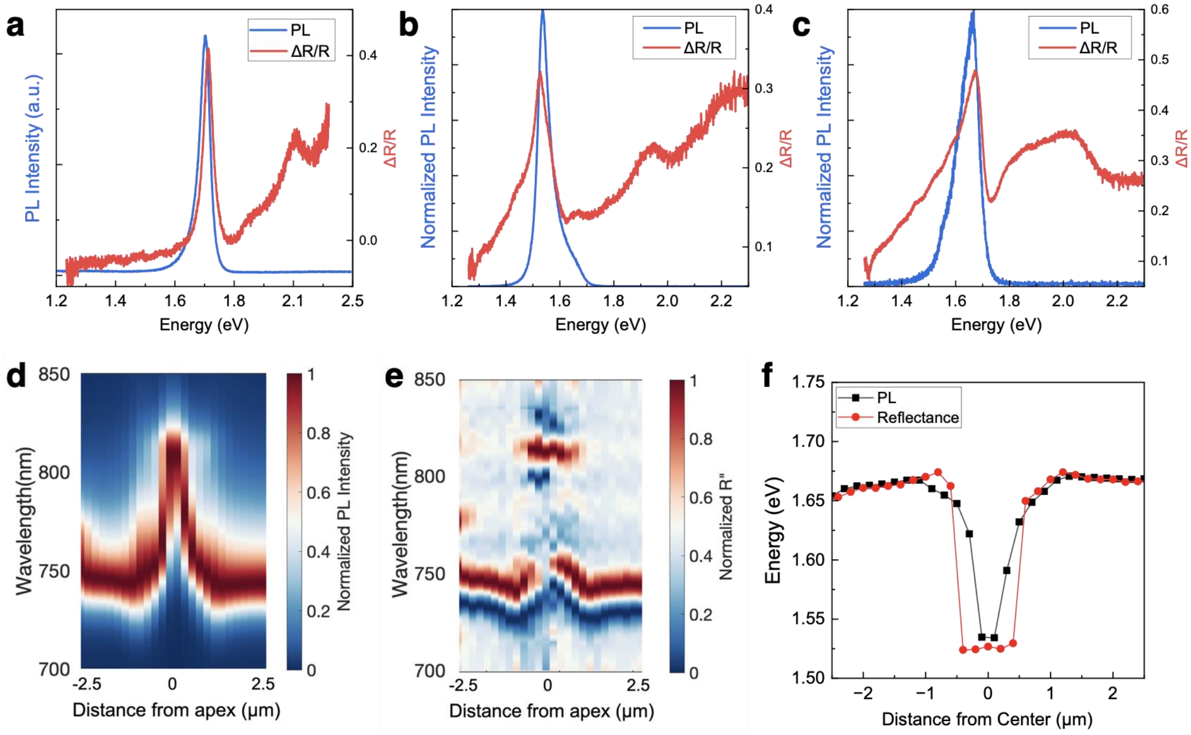

Supplementary Fig. 20. PL and differential reflectance (ΔR/R) spectra obtained from (a) flat WSe_2, (b) wrinkled WSe_2 at apex, and (c) wrinkled WSe_2 at valley. d-e, Hyperspectral line scan of (a) PL and (b) second derivative of differential reflectance across wrinkle structure. f, Modulation of exciton energy measured by hyperspectral imaging.

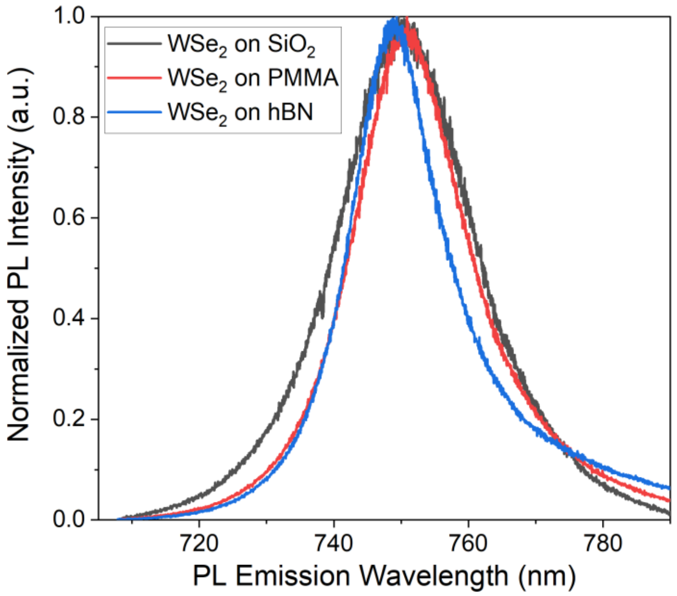

Supplementary Fig. 21. PL emission spectra of WSe_2 on various substrates. Flat WSe_2 is on bare 300 nm SiO_2/Si wafer, 70 nm-thick PMMA spincoated on SiO_2/Si wafer, and h-BN exfoliated on SiO_2/Si wafer.

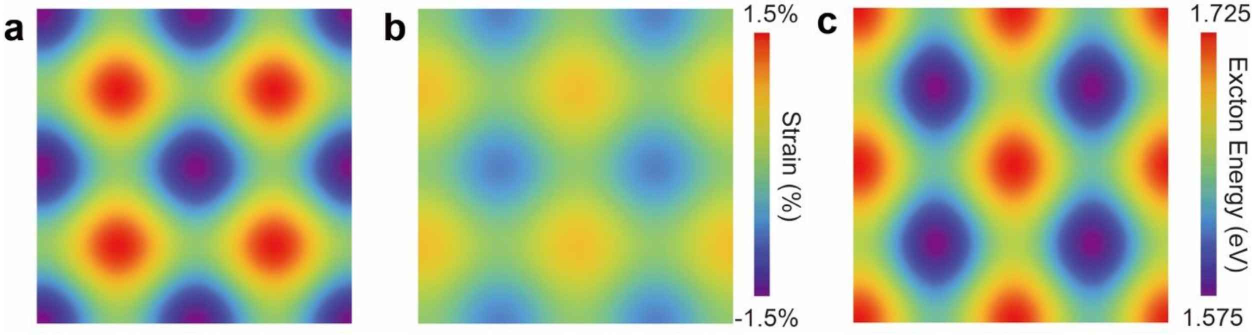

Supplementary Fig. 22. Strain distribution and exciton energy modulation in the checkerboard shape wrinkle model. a, Distribution of direct strain component ($\varepsilon_{xx} + \varepsilon_{yy}$). **b**, Distribution of shear strain component. **c**, Exciton energy modulated by direct and shear strain components.

Reference

1. Rhee, D., Lee, W. K. & Odom, T. W. Crack-Free, Soft Wrinkles Enable Switchable Anisotropic Wetting. *Angewandte Chemie - International Edition* **56**, 6523–6527 (2017).
2. Castellanos-Gomez, A. *et al.* Local strain engineering in atomically thin MoS2. *Nano Lett* **13**, 5361–5366 (2013).
3. Vella, D., Bico, J., Boudaoud, A., Roman, B. & Reis, P. M. The macroscopic delamination of thin films from elastic substrates. *Proc Natl Acad Sci U S A* **106**, 10901–10906 (2009).
4. Hu, G. *et al.* Controlling the Dirac point voltage of graphene by mechanically bending the ferroelectric gate of a graphene field effect transistor. *Mater Horiz* **6**, 302–310 (2019).
5. Mohiuddin, T. M. G. *et al.* Uniaxial strain in graphene by Raman spectroscopy: G peak splitting, Grüneisen parameters, and sample orientation. *Phys Rev B Condens Matter Mater Phys* **79**, 205433 (2009).
6. Conley, H. J. *et al.* Bandgap engineering of strained monolayer and bilayer MoS2. *Nano Lett* **13**, 3626–3630 (2013).
7. Desai, S. B. *et al.* Strain-induced indirect to direct bandgap transition in multilayer WSe2. *Nano Lett* **14**, 4592–4597 (2014).
8. Schmidt, R. *et al.* Reversible uniaxial strain tuning in atomically thin WSe2. *2d Mater* **3**, 021011 (2016).
9. Island, J. O. *et al.* Precise and reversible band gap tuning in single-layer MoSe2 by uniaxial strain. *Nanoscale* **8**, 2589–2593 (2016).
10. Li, Z. *et al.* Efficient strain modulation of 2D materials via polymer encapsulation. *Nat Commun* **11**, 1151 (2020).
11. Nelson, A. Co-refinement of multiple-contrast neutron/X-ray reflectivity data using MOTOFIT. *J Appl Crystallogr* **39**, 273–276 (2006).
12. Wang, G. *et al.* In-Plane Propagation of Light in Transition Metal Dichalcogenide Monolayers: Optical Selection Rules. *Phys Rev Lett* **119**, 047401 (2017).
13. You, Y. *et al.* Observation of biexcitons in monolayer WSe 2. *Nat Phys* **11**, 477–481 (2015).
14. Arora, A. *et al.* Excitonic resonances in thin films of WSe2: From monolayer to bulk material. *Nanoscale* **7**, 10421–10429 (2015).
15. Cai, T. *et al.* Coupling Emission from Single Localized Defects in Two-Dimensional Semiconductor to Surface Plasmon Polaritons. *Nano Lett* **17**, 6564–6568 (2017).
16. Li, H. & Zhang, X. H. Temperature-dependent photoluminescence and time-resolved photoluminescence study of monolayer molybdenum disulfide. *Opt Mater (Amst)* **107**, (2020).
17. Ross, J. S. *et al.* Electrical control of neutral and charged excitons in a monolayer semiconductor. *Nat Commun* **4**, (2013).
18. Gelly, R. J. *et al.* Probing dark exciton navigation through a local strain landscape in a WSe2 monolayer. *Nat Commun* **13**, (2022).
19. McCreary, K. M., Hanbicki, A. T., Sivaram, S. V. & Jonker, B. T. A- and B-exciton photoluminescence intensity ratio as a measure of sample quality for transition metal dichalcogenide monolayers. *APL Mater* **6**, (2018).
20. Aslan, O. B., Deng, M. & Heinz, T. F. Strain tuning of excitons in monolayer WSe2. *Phys Rev B* **98**, 115308 (2018).

21. Wang, G. *et al.* In-Plane Propagation of Light in Transition Metal Dichalcogenide Monolayers: Optical Selection Rules. *Phys Rev Lett* **119**, 047401 (2017).
22. You, Y. *et al.* Observation of biexcitons in monolayer WSe2. *Nat Phys* **11**, 477–481 (2015).
23. Arora, A. *et al.* Excitonic resonances in thin films of WSe2: From monolayer to bulk material. *Nanoscale* **7**, 10421–10429 (2015).
24. Cai, T. *et al.* Coupling Emission from Single Localized Defects in Two-Dimensional Semiconductor to Surface Plasmon Polaritons. *Nano Lett* **17**, 6564–6568 (2017).
25. Zhao, W. *et al.* Evolution of electronic structure in atomically thin sheets of ws 2 and wse2. *ACS Nano* **7**, (2013).
26. Feng, J., Qian, X., Huang, C. W. & Li, J. Strain-engineered artificial atom as a broad-spectrum solar energy funnel. *Nat Photonics* **6**, 866–872 (2012).
27. Stier, A. V. *et al.* Magnetooptics of Exciton Rydberg States in a Monolayer Semiconductor. *Phys Rev Lett* **120**, 957405 (2018).
28. Cadiz, F. *et al.* Exciton diffusion in WSe2 monolayers embedded in a van der Waals heterostructure. *Appl Phys Lett* **112**, 152106 (2018).
29. Ishii, A., Machiya, H. & Kato, Y. K. High Efficiency Dark-to-Bright Exciton Conversion in Carbon Nanotubes. *Phys Rev X* **9**, 041048 (2019).
30. Yuan, L. & Huang, L. Exciton dynamics and annihilation in WS2 2D semiconductors. *Nanoscale* **7**, 7402–7408 (2015).
31. Mouri, S. *et al.* Nonlinear photoluminescence in atomically thin layered WSe2 arising from diffusion-assisted exciton-exciton annihilation. *Phys Rev B Condens Matter Mater Phys* **90**, 155449 (2014).
32. Cordovilla Leon, D. F., Li, Z., Jang, S. W., Cheng, C. H. & Deotare, P. B. Exciton transport in strained monolayer WSe2. *Appl Phys Lett* **113**, 252101 (2018).
33. Niehues, I. *et al.* Strain Control of Exciton-Phonon Coupling in Atomically Thin Semiconductors. *Nano Lett* **18**, 1751–1757 (2018).
34. Hosseini, M., Elahi, M., Pourfath, M. & Esseni, D. Very large strain gauges based on single layer MoSe2 and WSe2 for sensing applications. *Appl Phys Lett* **107**, 253503 (2015).
35. Peng, G. H. *et al.* Distinctive Signatures of the Spin- and Momentum-Forbidden Dark Exciton States in the Photoluminescence of Strained WSe 2 Monolayers under Thermalization. *Nano Lett* **19**, 2299–2312 (2019).
36. Spataru, C. D., Ismail-Beigi, S., Capaz, R. B. & Louie, S. G. Theory and Ab initio calculation of radiative lifetime of excitons in semiconducting carbon nanotubes. *Phys Rev Lett* **95**, 247402 (2005).
37. Robert, C. *et al.* Fine structure and lifetime of dark excitons in transition metal dichalcogenide monolayers. *Phys Rev B* **96**, 155423 (2017).
38. Deilmann, T. & Thygesen, K. S. Finite-momentum exciton landscape in mono- and bilayer transition metal dichalcogenides. *2d Mater* **6**, 035003 (2019).
39. Stier, A. V., Wilson, N. P., Clark, G., Xu, X. & Crooker, S. A. Probing the Influence of Dielectric Environment on Excitons in Monolayer WSe2: Insight from High Magnetic Fields. *Nano Lett* **16**, 7054–7060 (2016).
40. Malyi, O. I. *et al.* Volume dependence of the dielectric properties of amorphous SiO2. *Physical Chemistry Chemical Physics* **18**, 7483–7489 (2016).

41. Hsu, W. T. *et al.* Dielectric impact on exciton binding energy and quasiparticle bandgap in monolayer WS2 and WSe2. *2d Mater* **6**, 025028 (2019).
42. Zeng, X. & Jiang, H. Strain effect of the dielectric constant in silicon dioxide. *Journal Of Microelectromechanical Systems* **20**, 353–354 (2011).
43. Falin, A. *et al.* Mechanical properties of atomically thin boron nitride and the role of interlayer interactions. *Nat Commun* **8**, 15815 (2017).
44. Chang, J., Toga, K. B., Paulsen, J. D., Menon, N. & Russell, T. P. Thickness Dependence of the Young's Modulus of Polymer Thin Films. *Macromolecules* **51**, 6764–6770 (2018).
45. Pellegrino, L., Khodaparast, S. & Cabral, J. T. Orthogonal wave superposition of wrinkled, plasma-oxidised, polydimethylsiloxane surfaces. *Soft Matter* **16**, 595–603 (2020).
46. Cai, S., Breid, D., Crosby, A. J., Suo, Z. & Hutchinson, J. W. Periodic patterns and energy states of buckled films on compliant substrates. *J Mech Phys Solids* **59**, 1094–1114 (2011).
47. Chen, X. & Hutchinson, J. W. Herringbone buckling patterns of compressed thin films on compliant substrates. *Journal of Applied Mechanics, Transactions ASME* **71**, 597–603 (2004).
48. Chen, X. & Hutchinson, J. W. A family of herringbone patterns in thin films. *Scr Mater* **50**, 797–801 (2004).
49. Li, H. *et al.* Optoelectronic crystal of artificial atoms in strain-textured molybdenum disulphide. *Nat Commun* **6**, 7381 (2015).
50. Rowland, R. Jr. *Principles of Solid Mechanics.* (CRC Press, 2019).
51. Aas, S. & Bulutay, C. Strain dependence of photoluminescence and circular dichroism in transition metal dichalcogenides: a k · p analysis. *Opt Express* **26**, 28672 (2018).
52. Reddy, J. N. *Theory and Analysis of Elastic Plates and Shells*. (CRC Press, 2006).
53. Mitchell, N. P. *et al.* Conforming nanoparticle sheets to surfaces with Gaussian curvature. *Soft Matter* **14**, 9107–9117 (2018).
54. Liu, X. & Guo, W. Shear strain tunable exciton dynamics in two-dimensional semiconductors. *Phys Rev B* **99**, 035401 (2019).
